# Comprehensive Liquid Biopsy Approaches for the Clinical Management of Lung Cancer Using Multiple Biological Matrices

**DOI:** 10.3390/ijms262311304

**Published:** 2025-11-22

**Authors:** Areti Strati, Martha Zavridou, Kostas A. Papavassiliou, Athanasios G. Papavassiliou

**Affiliations:** 1 Department of Biological Chemistry, Medical School, National and Kapodistrian University of Athens, 11527 Athens, Greece; papavas@med.uoa.gr; 2 Department of Genitourinary Medical Oncology, The University of Texas MD Anderson Cancer Center, Houston, TX 77030, USA; mzavridou@mdanderson.org; 3 First University Department of Respiratory Medicine, ‘Sotiria’ Chest Hospital, Medical School, National and Kapodistrian University of Athens, 11527 Athens, Greece; konpapav@med.uoa.gr

**Keywords:** liquid biopsy, lung cancer, biomarkers

## Abstract

Lung cancer is the most commonly diagnosed cancer and the leading cause of cancer-related mortality in both men and women. It is broadly classified into two main histological subtypes, with non-small cell lung cancer (NSCLC) being the most prevalent, accounting for approximately 85–90% of all cases. Liquid biopsy refers to the analysis of tumor-derived material circulating in body fluids. This minimally invasive technique can be performed repeatedly over time and enables the detection of a tumor’s genomic profile without tissue samples. Liquid biopsies have the potential to identify biomarkers across different lung cancer subtypes that may be associated with early detection, prognosis, and prediction of response to targeted therapies. In this context, bioinformatics tools play a critical role in analyzing large-scale, high-dimensional omics datasets, which can be transformed into clinically meaningful insights. This article emphasizes the significance of prognostic, predictive, and diagnostic biomarkers in lung cancer, which can be detected in various biological fluids. Furthermore, it highlights how integrating bioinformatics approaches can facilitate the development of a personalized molecular profile, ultimately supporting individualized treatment strategies for each patient.

## 1. Introduction

Lung cancer was the most commonly diagnosed malignancy in 2022, accounting for nearly 2.5 million new cases—approximately one in every eight cancer diagnoses worldwide—followed by breast, colorectal, prostate, and stomach cancers [1]. Lung cancer is classified into two major types: small cell lung cancer (SCLC) and non-small cell lung cancer (NSCLC), with NSCLC being the predominant subtype, representing approximately 85–90% of all lung cancer cases [2]. Treatment options for lung cancer depend on the type and stage of the disease and include surgery, chemotherapy, radiotherapy, targeted therapy, and immunotherapy [3].

Liquid biopsy is a minimally invasive technique involving the analysis of tumor-derived material circulating in body fluids, either as an alternative to or in addition to a solid tumor biopsy. It can be performed repeatedly over time and offers real-time insights into tumor heterogeneity, which may not be fully captured by a single tissue biopsy [4]. Combined analysis of tissue and liquid biopsy samples can provide complementary information by delineating clonal architecture in advanced cancer [5], detecting minimal residual disease (MRD) when genomic alterations are undetectable in tissue samples, or enhancing the identification of actionable gene rearrangements confirmed through next-generation sequencing (NGS) [6]. Moreover, liquid biopsy enables the characterization of genomic profiles in the absence of tissue, supporting improved patient management and longitudinal monitoring of disease progression and clonal evolution [7].

**Figure 1:**
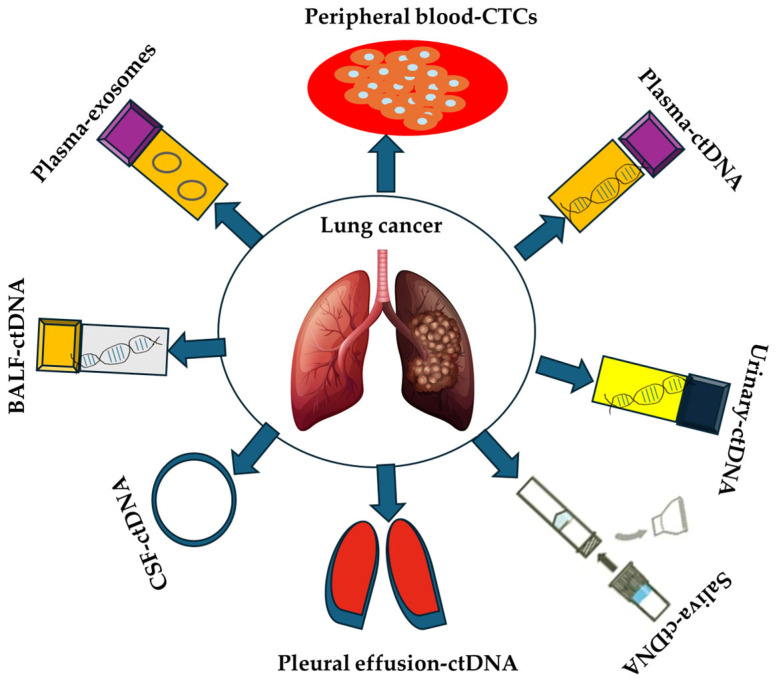
Use of alternative fluids in the liquid biopsy of lung cancer patients.

Numerous studies have applied liquid biopsy across various lung cancer subtypes to identify biomarkers associated with early detection, prognosis—either favorable or unfavorable—and treatment response or resistance [8,9,10]. Among these, the epidermal growth factor receptor (EGFR) is the most extensively studied biomarker in lung cancer, encompassing multiple mutations that assist clinicians in optimizing therapeutic decisions, including the initiation [5,11] or discontinuation of targeted treatments [12]. Predictive biomarkers, in particular, can determine whether a patient is likely to respond to targeted therapies, chemotherapy, immunotherapy, or other treatment modalities [13]. This review underscores the clinical significance of prognostic, predictive, and diagnostic biomarkers in lung cancer that can be detected in a variety of biological fluids through liquid biopsy (Figure 1).

## 2. Peripheral Blood

### 2.1. Plasma

#### 2.1.1. ctDNA Biomarkers of Prognostic Significance

##### Mutation Analysis

**Figure 2:**
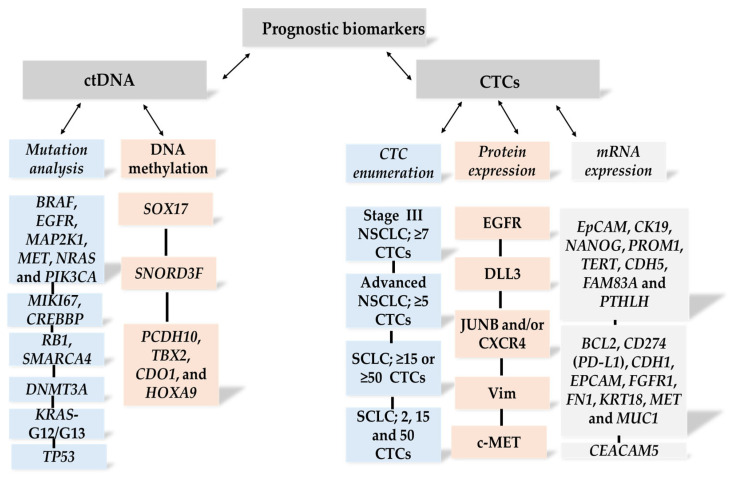
Overview of prognostic biomarkers derived from liquid biopsies in lung cancer patients.

Gene mutations associated with lung cancer include *EGFR*, *HER2*, and other oncogenic drivers, such as *KRAS*, *ALK*, *TP53*, and *MET* [14]. A growing body of evidence has demonstrated the prognostic and therapeutic significance of detecting these mutations in lung cancer patients, particularly *EGFR* mutations (Figure 2).

Osimertinib, a third-generation EGFR-tyrosine kinase inhibitor (EGFR-TKI), has been shown to be effective in NSCLC tumors exhibiting allele frequencies of *EGFR*-activating and/or *EGFR*-resistance mutations in plasma circulating tumor DNA (ctDNA), which are independently associated with poorer survival outcomes [15].

In patients without acquired *EGFR* resistance mutations, alterations in exon 545 of the *PIK3CA* gene represent the most common genetic events associated with disease progression [16]. Furthermore, in resectable NSCLC patients presenting with locoregional recurrence (LR), ctDNA analyses have revealed the coexistence of *TP53* and *EGFR* mutations [17]. A positive baseline ctDNA level has also been identified as an independent risk factor for disease-free survival (DFS), along with stage and micropapillary subtype, in patients with curatively resected *EGFR*-mutated stage I–IIIA NSCLC [18].

In a large cohort of NSCLC patients, 1688 somatic mutations were detected across 473 ctDNA samples. Survival analysis revealed that overall survival (OS) was significantly shorter in patients harboring *BRAF*, *PIK3CA*, and *KRAS* mutations [19]. Notably, NSCLC with *HER2* alterations is now recognized as a distinct molecular subtype [20]. In advanced NSCLC cases with *HER2* mutations, genomic profiling of ctDNA has shown that *HER2* alterations are detectable in almost all patients. The concurrent detection of *TP53* and *DNMT3A* mutations at baseline, coupled with persistent *HER2* mutations after initial therapy, has been correlated with an unfavorable prognosis [21].

Moreover, in advanced NSCLC, the assessment of 73 hotspot mutations in *EGFR*, *KRAS*, *BRAF*, *ERBB2*, and *PIK3CA* demonstrated that a decrease in ctDNA levels 4–6 weeks after treatment initiation was associated with prolonged median progression-free survival (PFS) and OS [22]. The prognostic value of ctDNA in advanced NSCLC has been further substantiated both before and after surgical resection, as preoperative ctDNA positivity serves as a strong predictor of shorter relapse-free survival (RFS) and OS, whereas postoperative ctDNA detection increases the likelihood of identifying early relapse [23].

Lung adenocarcinoma (LUAD), in particular, necessitates the identification of novel biomarkers and therapeutic targets to overcome its high mortality rate and limited treatment efficacy [24]. Recent evidence indicates that detection of *KRAS* mutations in resectable LUAD is associated with a more advanced disease stage and poorer survival outcomes [25].

Emerging data suggest that tissue tumor mutational burden (TMB) serves as a promising biomarker for predicting responses to PD-1/PD-L1 inhibitors, with a threshold of at least 10 mutations per megabase [26]. However, in a small NSCLC cohort treated with immune checkpoint inhibitors (ICIs), higher ctDNA TMB paradoxically predicted worse clinical outcomes [27]. In another study utilizing targeted NGS of plasma ctDNA from 50 patients with advanced NSCLC undergoing immunotherapy, reductions in ctDNA levels were associated with improved outcomes. Among the analyzed genes, *TP53* and *KRAS* were the most frequently mutated, while *BRAF*, *EGFR*, *MAP2K1*, *MET*, *NRAS*, and *PIK3CA* mutations were detected at different time points [28].

In patients with advanced NSCLC who derived sustained benefit (>12 months) from PD-(L)1 blockade, ctDNA dynamics have been shown to predict the risk of eventual progression [29]. Furthermore, combination strategies involving antiangiogenic therapy and ICI appear to normalize aberrant vascular–immune crosstalk and enhance the efficacy of immunotherapy [30]. In this context, ctDNA detection of *MIKI67* or *CREBBP* mutations in patients receiving combined antiangiogenic/ICI therapy has been associated with poorer PFS compared to mutation-negative cases [31]. Similarly, TMB analysis of ctDNA plasma samples from NSCLC patients treated with either immune monotherapy or combination therapy revealed that RB1 and *SMARCA4* mutations were strongly correlated with inferior survival outcomes [32].

At the genomic stability level, DNA double-strand breaks (DSBs) contribute significantly to tumorigenesis by promoting genomic instability, while DSB repair mechanisms serve as key pathways for tumor suppression [33]. In early-stage NSCLC patients without prior therapy, increased genomic instability related to DNA interstrand cross-linking and DSB repair processes has been significantly associated with early tumor recurrence [34]. Furthermore, the presence of sensitizing *EGFR* mutations prior to radical radiotherapy has also been linked to an increased likelihood of early recurrence [35].

Comprehensive tumor exome sequencing of ctDNA samples from early-stage NSCLC patients identified somatic mutations whose detection prior to treatment correlated with shorter OS and RFS [36]. Similarly, post-treatment detection of ctDNA was found to be associated with shorter survival outcomes [37], while preoperative detection of ctDNA has been consistently shown to be significantly associated with shorter recurrence-free survival [38]. Importantly, integration of radiologic imaging with ctDNA assessment, both at baseline and following treatment, enables stratification of patients into risk groups that more accurately predict clinical outcomes [39].

**Figure 3:**
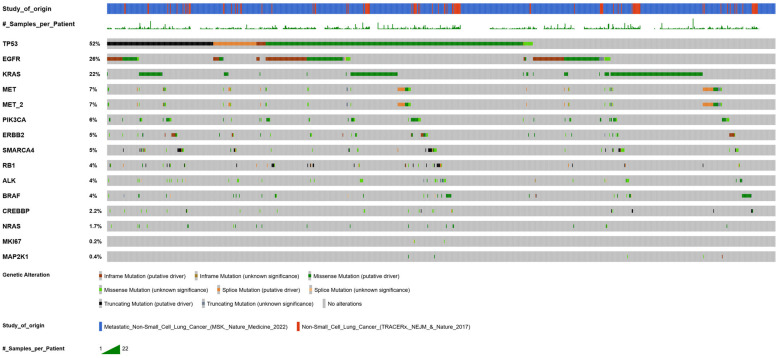
Overview of the mutational landscape in ctDNA isolated from patients with NSCLC. (Data accessed via the cBioPortal for Cancer Genomics (https://www.cbioportal.org/, accessed on 10 November 2025).

A graphical representation from cBioPortal illustrating TP53 and other key mutations within the mutational landscape is shown in Figure 3 (Data accessed via the cBioPortal for Cancer Genomics (https://www.cbioportal.org/, accessed on 10 November 2025), combining data from two independent studies [40,41].

##### DNA Methylation

DNA methylation is a fundamental epigenetic mechanism implicated in the pathogenesis and progression of lung cancer, leading to aberrant gene expression that facilitates tumor initiation and evolution [42]. Distinct methylation landscapes have been observed across various lung cancer subtypes [43], while specific methylation profiles have been identified in tumors harboring mutations in the *EGFR*, *KRAS*, or *TP53* genes [44]. These findings collectively underscore the role of epigenetic dysregulation as a driver of molecular heterogeneity in lung cancer.

The methylation status of the *SOX17* promoter has been investigated in ctDNA isolated from patients with advanced NSCLC, demonstrating an independent prognostic value with respect to OS [45]. Moreover, a novel approach to detect *ALK*-specific 5-methylcytosine (5-mC) alterations in cell-free DNA (cfDNA) has recently been described. The resulting 5-mC–based tumor DNA score (5-mC score) showed a strong correlation with chromosomal instability and *EML4*-*ALK* fusion frequency in cfDNA samples. Importantly, patients with high 5-mC scores exhibited significantly shorter OS compared with those with low scores [46].

Furthermore, methylation profiling of ctDNA in patients with advanced NSCLC receiving EGFR-TKIs revealed that hypermethylation of the *SNORD3F* locus was associated with the poorest clinical outcomes. Elevated *SNORD3F* methylation levels were also identified as an independent risk factor for disease recurrence, highlighting the potential utility of methylation-based biomarkers for prognostic stratification and therapeutic monitoring in NSCLC [47].

#### 2.1.2. ctDNA Biomarkers of Predictive Significance

Liquid biopsy enables the identification of predictive biomarkers and represents a paradigm shift toward more personalized and less invasive cancer management, fundamentally transforming the landscape of precision oncology [48]. In lung cancer, predictive biomarkers detected through ctDNA include *EGFR* mutations that guide targeted therapy selection, blood tumor mutational burden (bTMB), and mutational signatures that predict responsiveness to immunotherapy, as well as the detection of MRD following treatment, which serves as a predictor of disease recurrence and survival outcomes [49].

**Figure 4:**
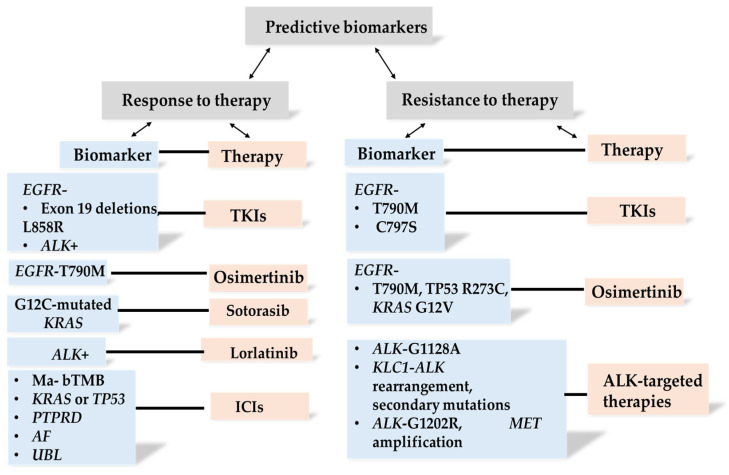
Overview of predictive biomarkers derived from liquid biopsies in lung cancer patients.

Collectively, predictive biomarkers play a pivotal role in distinguishing patients who are likely to benefit from specific therapeutic interventions from those who may develop resistance. The principal categories and clinical implications of these ctDNA-based predictive biomarkers are outlined in detail below (Figure 4).

##### Response to Therapy

Mutations in the *EGFR* gene play a pivotal role in the development and progression of lung cancer, particularly in NSCLC, the most prevalent histological subtype. The most common oncogenic *EGFR* alterations, including exon 19 deletions and the L858R point mutation, confer sensitivity to targeted therapy with tyrosine kinase inhibitors (TKIs) [50]. However, osimertinib has demonstrated high efficacy in NSCLC patients harboring the T790M resistance mutation detected through ctDNA, even in cases where tumor mutation status is unknown and progression has occurred following prior EGFR-TKI therapy [51].

Complete clearance of *EGFR* mutations from plasma following osimertinib administration in advanced NSCLC has been shown to strongly predict PFS, objective response rate (ORR), and disease control rate (DCR) [52]. Similarly, in patients with *EGFR*-mutated NSCLC, the clearance of ctDNA-detectable mutations during first-line TKI treatment has been identified as a robust predictor of a favorable outcome [53]. Continuous monitoring of variant allele frequency (VAF) in serial plasma samples from advanced NSCLC patients has revealed distinct molecular response and progression patterns. A reduction or complete disappearance of mutant alleles correlates with clinical response, whereas the emergence or increase in new variants reflects disease progression, thus providing a reliable molecular tool for treatment monitoring [54]. Moreover, the absence of *EGFR* copy number gain detected in ctDNA has been associated with exceptional, long-term responses (>3 years) in NSCLC patients treated with afatinib [55].

Comprehensive genomic profiling of blood-derived ctDNA from patients with NSCLC adenocarcinoma has identified frequent alterations in *TP53*, *EGFR*, *MET*, *KRAS*, and *ALK* genes. Patients who subsequently received genotype-matched therapies exhibited high rates of stable disease (SD ≥ 6 months) or partial response (PR) [56]. Another clinically relevant target is the *KRAS* G12C mutation, which can be irreversibly inhibited by sotorasib. Early clearance of *KRAS* G12C–mutated ctDNA has been shown to predict clinical benefit in patients with advanced NSCLC treated with sotorasib [57]. Similarly, detection of the *BRAF* V600E mutation in plasma samples from newly diagnosed NSCLC patients enables the use of *BRAF*-targeted therapies [58].

In plasma DNA from *ALK*-positive (*ALK*^+^) NSCLC patients receiving TKIs, integration of copy number variation (CNV) profiling with targeted panel sequencing has improved the molecular monitoring of disease, particularly in high-risk or heavily pretreated cases [59]. Administration of lorlatinib in *ALK*^+^ NSCLC was shown to markedly reduce delta variant allele frequency (dVAF ≤ 0), correlating with prolonged PFS [60]. In SCLC, dynamic and sustained clearance of total circulating tumor load (cfTL) following systemic chemotherapy or immunotherapy can be robustly monitored through ctDNA sequence and structural analysis, even prior to radiographic progression [61].

Retrospective studies have consistently suggested that tumors with a high TMB exhibit greater sensitivity to immune checkpoint inhibitors (ICIs) [62,63,64,65]. Conversely, whole-exome sequencing (WES) of ctDNA from advanced NSCLC patients treated with ICIs revealed that durable responders tended to have low bTMB [66]. Notably, maximum somatic allele frequency (MSAF)-matched bTMB (Ma-bTMB) has been proposed as a superior predictive metric, identifying more patients who may benefit from ICI therapy compared with standard bTMB or low allele frequency (LAF)-bTMB, in combination with PD-L1 expression levels [67].

Beyond TMB, limited gene panels have also demonstrated predictive power for immunotherapy response using algorithms such as the “high immune score.” The absence of targetable driver mutations (*EGFR*, *ROS1*, *ALK*, *BRAF V600E*) and of *PTEN* or *STK11* mutations, combined with the presence of *KRAS* or *TP53* transversion mutations, has been correlated with favorable ICI response in advanced NSCLC [68]. In non-squamous NSCLC, detection of deleterious mutations within the *PTPRD* phosphatase domain in ctDNA has emerged as a predictive biomarker identifying patients who derive greater benefit from ICI therapy than from chemotherapy, regardless of TMB, PD-L1 status, or *TP53*/*KRAS*/*EGFR* mutation background [69].

Dynamic monitoring of ctDNA during immunotherapy provides additional predictive insights. In metastatic NSCLC patients receiving ICIs, a reduction in ctDNA levels closely mirrors radiologic response, suggesting that ctDNA decline serves as an early marker of therapeutic efficacy [66]. Similarly, reductions in allele frequency (AF) within two weeks of nivolumab initiation have been strongly associated with durable responses [70]. Conversely, rising MAF values in the presence of co-mutations such as *BRAF* V600E, *KRAS* G12V, or *TP53* M237I may serve as early indicators of molecular and radiologic progression [71].

Indeed, VAF alterations may precede radiologic changes. For example, Raja et al. demonstrated that fluctuations in ctDNA VAF strongly correlated with the long-term benefit of durvalumab (anti–PD-L1 therapy) [72]. Similarly, a rapid decline in ctDNA AF between baseline and early post-treatment blood samples during first-line pembrolizumab therapy was predictive of clinical benefit in advanced NSCLC [73]. Parallel findings have shown that decreases in ctDNA levels after two cycles of ICI treatment (t0 → t1) occur predominantly among responders [74]. Finally, ubiquitin-like conjugation (*UBL*) gene mutations have recently been investigated as potential biomarkers for response stratification to second-line immunotherapy in NSCLC, demonstrating particularly strong predictive value among *TP53* wild-type subgroups [75].

##### Resistance to Therapy

The “gatekeeper” mutation T790M within the *EGFR* kinase domain—where a methionine residue at position 790 sterically hinders inhibitor binding—confers resistance to most first- and second-generation EGFR-TKIs [76]. Quantification of circulating tumor DNA (ctDNA) levels has emerged as a powerful tool for detecting newly acquired resistance mutations in NSCLC patients receiving first-line TKI therapy. Agulnik et al. demonstrated that dynamic monitoring of the clearance and re-emergence of driver mutations during TKI treatment enables the early detection of disease progression [77].

Genome-wide somatic copy number alterations (SCNAs) identified in ctDNA from plasma samples of patients with advanced *EGFR*-mutated lung adenocarcinoma represent another mechanism of resistance, particularly to third-generation TKIs such as osimertinib, which are associated with reduced therapeutic response rates [78]. The unusual mutations *TP53* R273C and *KRAS* G12V have been detected in advanced NSCLC patients with *EGFR* T790M mutation, which attenuates the efficacy of osimertinib [79]. In addition, the *EGFR* C797S resistance mutation has been shown to render sunvozertinib, an EGFR-TKI targeting exon 20 insertions, ineffective [80].

Molecular analysis of plasma-derived DNA/RNA in patients with advanced NSCLC has revealed de novo mutations that emerge during disease progression and contribute to treatment resistance, as reported by Lupini et al., who identified 13 such alterations [81]. Consequently, ctDNA profiling serves as a crucial approach for elucidating signaling pathway disruptions and uncovering mechanisms of therapeutic resistance, thereby facilitating the development of next-generation targeted therapies [82,83].

Acquired secondary *ALK* mutations have also been implicated in resistance to ALK-targeted therapies. For instance, the *ALK* G1128A mutation was detected via NGS in the ctDNA of an *ALK*-positive (*ALK*^+^) NSCLC patient who experienced disease progression after a brief partial response to crizotinib, highlighting the utility of liquid biopsy in identifying resistance mechanisms [84]. Similarly, in an NSCLC patient harboring a *KLC1*-*ALK* rearrangement, four distinct secondary *ALK* mutations were identified in ctDNA at the time of progression, all potentially contributing to therapeutic failure [85].

Increased detection of *ALK* resistance mutations has been shown to correlate with the high-risk clinical phenotype associated with the *EML4*-*ALK* variant 3 (V3), as confirmed by liquid biopsy analyses in *ALK*^+^ NSCLC patients [86]. Serial ctDNA analyses have further revealed that *MET* amplification and *ALK* G1202R substitution constitute major mechanisms of acquired resistance to ALK inhibitors [87]. Identification of various putative *ALK* inhibitor (ALK-I) resistance mutations thus provides a valuable framework for optimizing treatment decisions [88]. For example, brigatinib, a next-generation selective ALK inhibitor, has demonstrated substantial activity in *ALK*-positive NSCLC pretreated with crizotinib, particularly in cases exhibiting ALK-dependent resistance mechanisms [89].

The *BRAF* V600E mutation, which defines a distinct molecular subset of NSCLC, constitutes an actionable therapeutic target for BRAF inhibitor monotherapy. Nevertheless, treatment with BRAF inhibitors alone is frequently associated with rapid acquisition of resistance [90]. ctDNA profiling in *BRAF*-mutated NSCLC has yielded significant insights into the molecular underpinnings of resistance to BRAF-targeted therapy (TT), implicating reactivation of the MAPK and PI3K signaling pathways as well as alterations in downstream signal transducers [91].

#### 2.1.3. ctDNA Biomarkers of Diagnostic Significance

##### miRNAs and Extracellular Vesicles (EVs)

Exosomes play a pivotal role in the pathogenesis and progression of lung cancer. Their ubiquitous presence in body fluids renders them a promising and minimally invasive source of diagnostic biomarkers [92]. Proteomic profiling of plasma-derived exosomal immunoglobulin subtypes, primarily related to antigen–antibody interactions, revealed significant differences in the expression of immunoglobulin heavy variable 4-4 (IGHV4-4) and immunoglobulin lambda variable 1-40 (IGLV1-40) between lung cancer patients and healthy controls, suggesting their potential as novel diagnostic indicators for NSCLC [93].

Furthermore, exosomal *PLA2G10* mRNA and its corresponding protein levels exhibit a strong positive correlation, and their combined evaluation has been associated with more aggressive NSCLC phenotypes, thereby enhancing diagnostic accuracy in distinguishing less malignant from more advanced disease [94]. Similarly, exosomal circRNAs such as circ_0061407 and circ_0008103 have been implicated in NSCLC progression through interactions with microRNAs and proteins [95].

Additional exosome-associated biomarkers have demonstrated value for the early detection of NSCLC, including elevated plasma GCC2 protein levels [96], significant downregulation of serum exosomal SNORD116 and SNORA21 [97], and reduced expression of miR-5684 and miR-125b-5p [98]. Moreover, several tRNA-derived fragments (tRFs)—tRF-Leu-TAA-005, tRF-Asn-GTT-010, tRF-Ala-AGC-036, tRF-Lys-CTT-049, and tRF-Trp-CCA-057—have been identified as potential diagnostic markers [99]. Likewise, dysregulated lncRNAs, including linc01125, HNF1A-AS1, MIR100HG, linc01160, and ZNRF3-AS1 [100], as well as plasma and exosomal mtDNA fragments (mtDNA79, mtDNA230, MTATP8) [101], and exosomal lncRNA RP5-977B1 [102], have shown promise for lung cancer detection. Elevated exosomal levels of α2-HS-glycoprotein (AHSG) and extracellular matrix protein 1 (ECM1) [103], along with decreased exosomal miR-620 expression [104], further underscore the diagnostic potential of exosomal components.

The diagnosis of lung cancer—including LUAD, lung squamous cell carcinoma (LUSC), and SCLC—may be facilitated by the detection of exosomal PITPNA-AS1, which is significantly upregulated in lung cancer patients and correlates strongly with tumor stage, lymph node involvement, and distant metastasis [105]. Additionally, a novel exosomal carbohydrate biomarker has been reported to be overexpressed in lung cancer patients relative to healthy individuals [106].

Comprehensive exosomal protein profiling has identified CD151, CD171, and tetraspanin 8 as robust diagnostic markers across lung cancer subtypes and disease stages [107]. Early detection of LUAD has been linked to increased CXCL7 protein levels in plasma exosomes [108], as well as upregulation of hsa-miR-4454, hsa-miR-619-5p [109], piR-hsa-26925, piR-hsa-5444 [110] miR-103b, miR-877-5p, and miR-29c-5p [111] and elevated serum exosomal miR-1290 [112].

Analysis of differentially expressed genes (DEGs) from The Cancer Genome Atlas (TCGA) revealed 1619 genes differentially expressed between LUSC and LUAD. Notably, the combined expression of tumor protein P63 (TP63), keratin 5 (KRT5), CEA cell adhesion molecule 6 (CEACAM6), and surfactant protein B (SFTPB) provided superior discriminatory power between LUSC and LUAD [113]. Significant differences in WNT5A expression were also observed between LUAD and LUSC subtypes [114].

Differentiation between LUAD and granuloma has been achieved using a six-exosomal-miRNA panel (miR-151a-5p, miR-30a-3p, miR-200b-5p, miR-629, miR-100, and miR-154-3p) [115]. Similarly, tumor-derived exosomal miRNAs can accurately distinguish between LUAD and LUSC in early-stage NSCLC: miR-181-5p, miR-30a-3p, miR-30e-3p, and miR-361-5p are adenocarcinoma-specific, whereas miR-10b-5p, miR-15b-5p, and miR-320b are squamous carcinoma-specific [116].

Exosomal miRNAs also represent promising non-invasive diagnostic biomarkers for metastatic NSCLC. Elevated expression of miR-320a, miR-622, and let-7f-5p [117], together with increased plasma exosomal levels of lipopolysaccharide-binding protein (LBP) [118], has been significantly correlated with metastatic disease. Furthermore, SCLC subtypes can be accurately identified by quantifying the mRNA expression of transcription factors *ASCL1*, *POU2F3*, and *NEUROD1* in exosome-rich EVs [119].

### 2.2. CTCs

#### 2.2.1. CTC Biomarkers of Prognostic Significance

Research in cancer biology provides critical insights into the fundamental mechanisms of metastasis and enables the identification of biomarkers that allow real-time monitoring of disease dynamics [120]. To date, the most significant prognostic biomarkers for cancer patients include circulating tumor cell (CTC) count, protein expression, and genomic profiling, as discussed below [10].

##### CTC Enumeration

Detection of CTCs has been explored even in the early stages of non-metastatic LUAD [121] and NSCLC [122], where their presence has been significantly associated with an increased risk of disease recurrence. In stage III NSCLC, a baseline CTC count of ≥7 prior to chemotherapy has been correlated with shorter progression-free survival (PFS) and overall survival (OS) [123]. Similarly, in advanced NSCLC, the presence of ≥5 CTCs has been established as an indicator of poor prognosis [124,125].

In SCLC, however, the optimal prognostic threshold for CTC enumeration remains controversial. Studies have reported that CTC counts of ≥15 or ≥50 are significantly associated with an elevated risk of disease progression [126], whereas Tay et al. demonstrated that thresholds of 2, 15, and 50 CTCs each show strong correlations with both PFS and OS. Importantly, dynamic changes in CTC count after a single chemotherapy cycle have been identified as an independent prognostic factor in SCLC [127].

CTC quantification also carries prognostic significance in advanced NSCLC patients treated with immunotherapy. Individuals with detectable CTCs exhibited significantly shorter PFS and OS compared to those without measurable CTCs [128].

Several studies have demonstrated that CTCs isolated from the pulmonary vein may serve as a particularly robust prognostic indicator in lung cancer [129,130]. Crosbie et al. reported that CTC counts in pulmonary venous blood were substantially higher than those in matched peripheral blood samples and represented an independent predictor of recurrence and mortality [131].

Beyond enumeration, the chromosomal ploidy of CTCs reflects the genetic heterogeneity of tumors. Aneuploidy and polyploidy contribute to chromosomal instability, enhancing karyotypic diversity and tumor evolution [132]. The presence of triploid and multiploid small CTCs, as well as tetraploid large CTCs, has been specifically associated with shorter OS in patients with advanced lung cancer [133].

##### Protein Expression

The expression of EGFR on circulating tumor cells (CTCs) in the peripheral blood of patients undergoing radical resection for NSCLC has been reported. Elevated EGFR expression in residual CTCs following surgery was significantly associated with early disease recurrence and shorter disease-free survival (DFS) [134]. Similarly, the presence of delta-like ligand 3 (DLL3)–positive CTCs at baseline in SCLC was significantly correlated with shorter progression-free survival (PFS) [135]. The transcription factors JUNB and CXCR4 were frequently detected in CTCs isolated from SCLC patients and have been linked to markedly worse overall survival (OS) [136].

In a multi-institutional prospective study, PD-L1 expression in peripheral circulating cells demonstrated prognostic relevance, as patients with >1.1 PD-L1(^+^) cells/mL exhibited significantly poorer OS compared with those with ≤1.1 PD-L1(^+^) cells/mL [137]. Consistent findings were reported by Sinoquet et al., who observed that the presence of PD-L1(^+^) CTCs was associated with inferior OS [138].

Epithelial–mesenchymal transition (EMT) plays a pivotal role in cancer metastasis by promoting tumor initiation and dissemination [139]. The expression of mesenchymal markers in CTCs has been extensively investigated, revealing that higher baseline counts of vimentin-positive (Vim^+^) CTCs and the presence of non-apoptotic CTCs in patients with progressive disease are independent prognostic factors associated with reduced OS [140]. EMT can also be induced through activation of the receptor tyrosine kinase c-MET. Interestingly, high expression of c-MET (c-METH) and low expression of E-cadherin (E-cadL) have been found to correlate with a more favorable prognosis in SCLC [141]. The interplay between PD-L1 expression and EMT in CTCs has also been investigated in NSCLC. The presence of more than three PD-L1^+^/EMT^+^ CTCs was significantly associated with poorer survival following curative surgery [142]. Similarly, among patients with advanced NSCLC receiving chemotherapy, those with >3 PD-1(^+^) CTCs at baseline exhibited a significantly shorter median PFS compared with patients with lower PD-1(^+^) CTC counts [143].

##### mRNA Expression

The expression of *CK19* mRNA has been extensively investigated in breast cancer, where it is associated with CTC malignancy and metastatic potential [144]. In metastatic NSCLC, the detection of *CK19* mRNA-positive CTCs before and after first-line chemotherapy has similarly been identified as an unfavorable prognostic indicator correlated with poor clinical outcomes [145].

Several additional transcripts, including *EpCAM*, *CK19*, *NANOG*, *PROM1*, *TERT*, *CDH5*, *FAM83A*, and *PTHLH* [146], as well as high expression levels of *BCL2*, *CD274* (*PD-L1*), *CDH1*, *EPCAM*, *FGFR1*, *FN1*, *KRT18*, *MET*, and *MUC1* [147], have been linked to reduced survival and aggressive tumor biology in NSCLC. Furthermore, the presence of *EpCAM*/*MUC1* mRNA-positive CTCs has been associated with shorter pre- and postoperative DFS and OS [148]. In metastatic NSCLC, persistent *CEACAM5* mRNA positivity both before and after therapy has been correlated with decreased PFS and OS [149].

In SCLC, mRNA expression of *DLL3*, a component of the Notch signaling pathway, has been reported as a marker of poor OS [150]. Similarly, elevated *PD-L1* mRNA expression in CTCs isolated from NSCLC patients during radiotherapy was shown to predict worse prognosis [151].

The folate receptor (FR) is a membrane-bound glycoprotein that facilitates high-affinity binding and transport of folate into cells [152]. In malignancies of epithelial origin, *FR-α* expression is frequently upregulated and correlates positively with tumor stage and grade [153]. In the context of liquid biopsy, *FR* mRNA expression has been explored as a prognostic biomarker; preoperative *FR^+^* CTC levels have been identified as a potential predictor of survival outcomes in NSCLC patients undergoing surgical resection [154].

#### 2.2.2. CTC Biomarkers of Predictive Significance

Although numerous biomarkers associated with poor clinical outcomes have been identified in CTCs, studies investigating predictive markers of response to specific therapies remain limited. The predictive value of CTC-derived biomarkers for immunotherapy response has been explored in advanced lung cancer prior to treatment initiation. In one study, the expression of *CEA*, *hTERT*, *CK19*, and *PD-L1* in CTCs was assessed, and only high expression levels of *CEA* and *hTERT* were significantly correlated with clinical response to nivolumab [155].

Monitoring dynamic changes in CTC counts during treatment represents a crucial parameter for assessing therapeutic efficacy and patient response. In primary lung cancer, variations in CTC counts after two cycles of chemotherapy were found to correlate with radiologic findings [156]. Similarly, in SCLC, a marked decrease in CTC levels after a single chemotherapy cycle predicted treatment response [157]. In NSCLC treated with pertuzumab and erlotinib, a strong correlation was observed between a reduction in CTC counts and radiologic response [158]. Using in vivo CTC isolation, Gorges et al. further demonstrated that therapeutic response in lung cancer was consistently associated with a significant decline in CTC counts during treatment [159].

Moreover, reductions in PD-L1^+^ CTCs have been observed among NSCLC patients responding to PD-1/PD-L1 immune checkpoint inhibitors [160]. In patients with *EGFR*-mutated advanced NSCLC receiving EGFR-TKI therapy, dynamic changes in CTC levels were significantly associated with both partial response and stable/progressive disease status [161]. Similarly, CTC enumeration may also serve as a predictive biomarker in *ALK*-rearranged NSCLC, offering valuable insights for the optimization of individualized therapeutic strategies in patients with advanced disease [162].

## 3. Bronchoalveolar Lavage (BAL) Fluid Biomarkers

Bronchoalveolar lavage (BAL) is a well-established diagnostic procedure in pulmonary medicine that involves the retrieval of fluid from the bronchoalveolar spaces of the lungs. It serves as a valuable tool for the diagnosis and characterization of various pulmonary diseases, providing key insights into underlying pathological processes and supporting the selection of appropriate therapeutic strategies [163]. BAL fluid contains cellular components and soluble proteins originating from the lower respiratory tract [163], while DNA purified from the BAL supernatant represents one of the richest sources of circulating tumor DNA (ctDNA) among respiratory samples [164].

A convenient and effective approach for detecting EGFR mutations in BAL-derived liquid biopsy samples from patients with lung adenocarcinoma has been reported. Park et al. demonstrated a high concordance of *EGFR* mutation status between tumor tissue and corresponding BAL samples [165]. Consistently, no statistically significant differences were observed in TMB or allele frequency heterogeneity (AFH) between BAL and tissue specimens [166]. When sequencing the mutational landscape of lung cancer using cell-free BAL fluid, a higher number of tumor-derived mutations with greater allele frequencies were identified compared to plasma-derived cell-free DNA [167]. Furthermore, *EGFR* mutation detection in BAL has shown higher sensitivity relative to venous blood samples [168].

Beyond genetic profiling, BAL is also a promising source of EVs suitable for genomic and proteomic analyses. EV-based evaluation of BAL fluid offers the advantage of reducing turnaround time for *EGFR* mutation confirmation, enabling earlier initiation of EGFR TKI therapy and potentially improving clinical outcomes [169].

Epigenetic alterations have also emerged as valuable biomarkers in BAL samples. DNA methylation signals detected in BAL fluid are derived from lung tissue, supporting their diagnostic potential [170]. In particular, *TMPRSS4* DNA methylation has been proposed as a molecular biomarker capable of refining outcome prediction across all stages of NSCLC. Hypomethylation of *TMPRSS4* may serve as a diagnostic indicator for early-stage NSCLC in both BAL and plasma samples, broadening the clinical utility of liquid biopsy-based assays [171].

Cytokine profiling in BAL fluid further enhances our understanding of the tumor microenvironment (TME) and the role of inflammation in lung cancer. Elevated levels of pro-inflammatory cytokines such as TNF-α in BAL and IL-6 in plasma have been correlated with disease recurrence [172]. Moreover, the detection of exhausted CD8^+^ T cells in the BAL of patients with advanced lung cancer suggests that T cells isolated from tumor-associated BAL (t-BAL) may represent an accessible surrogate for tumor-infiltrating lymphocytes [173].

Proteomic analyses of BAL samples have also demonstrated strong potential for improving early detection and molecular stratification of lung cancer. In a prospective cohort of 90 patients with suspected lung cancer followed for two years, Carvalho et al. identified 133 potential protein biomarkers that were differentially expressed between cancer and non-cancer cases [174]. Furthermore, 25 glycoproteins exhibited at least a twofold difference in expression between malignant and benign BAL samples [175]. Collectively, these findings underscore the growing clinical relevance of BAL fluid as a minimally invasive yet information-rich source for genomic, epigenomic, proteomic, and immunologic biomarker discovery in lung cancer.

## 4. Other Biological Fluids

### 4.1. Urine Samples

Beyond peripheral blood and BAL fluid, alternative biological fluids—such as urine and cerebrospinal fluid—have also been utilized for the detection of ctDNA. In a study involving 150 patients with NSCLC, analysis of cell-free DNA in urine revealed a strong correlation with treatment efficacy. Notably, the secondary *EGFR* resistance mutation T790M was also identified through this approach [176]. These findings suggest that ctDNA may be shed into the bloodstream and subsequently excreted into the urine, supporting the development of combinatorial strategies to address both inter- and intralesional tumor heterogeneity and to characterize minimal residual disease following initial therapy [177]. Furthermore, urinary ctDNA methylation analysis represents another promising avenue, as elevated methylation levels at specific gene promoters have been observed in NSCLC patients [178].

### 4.2. Saliva

Saliva has emerged as an additional non-invasive biofluid for the detection of ctDNA in patients with lung cancer. The *EGFR* L858R mutation has been successfully detected as ultra-short ctDNA (usctDNA)—typically 40–60 base pairs in length—in both plasma and saliva samples. Interestingly, the majority of these usctDNAs are primarily localized within the exosomal fraction, suggesting a potential mechanism of stabilization and transport in EVs [179].

### 4.3. Exhaled Breath Condensate (EBC)

Exhaled breath condensate (EBC) offers a lung-specific liquid biopsy modality with promising diagnostic potential. In lung cancer patients, EBC analysis has revealed a greater number of detectable mutations in key oncogenes such as *EGFR*, *KRAS*, and *PIK3CA* compared to matched tumor tissue samples. These results highlight the utility of EBC as a complementary source of molecular information, particularly when integrated with plasma-based assays [180].

### 4.4. Cerebrospinal Fluid (CSF)

Cerebrospinal fluid (CSF) constitutes a particularly valuable medium for the investigation of brain metastases in lung cancer. The combined analysis of plasma and CSF has been shown to enhance the diagnostic accuracy of *EGFR* genotyping in central nervous system (CNS) metastases [181] while also facilitating the monitoring of intracranial tumor evolution over time [182]. Notably, the analysis of CSF has led to the discovery of *ANKRD11*, a novel biomarker with significant prognostic implications [183]. Moreover, sequencing of ctDNA in CSF from patients with NSCLC and brain metastases has revealed distinct mutational patterns in driver genes [184]. Importantly, ctDNA derived from CSF appears to be superior to plasma ctDNA in comprehensively capturing the mutational landscape of brain metastases, as it enables the detection of all relevant mutations in the majority of patients [185]. In addition to ctDNA, circulating tumor cells (CTCs) have also been identified in CSF (referred to as CSF-CTCs), representing an alternative liquid biopsy approach for genomic profiling in NSCLC patients with leptomeningeal metastases [186].

### 4.5. Pleural Effusions (PE)

Pleural effusions (PE) have also been explored as a valuable source of ctDNA in patients with lung cancer. Evidence suggests that the supernatant of PE may exhibit a higher overall mutation rate compared to plasma, and a greater frequency of atypical genetic alterations has also been reported [187]. This highlights the potential of PE to serve as a complementary specimen to plasma in ctDNA analysis, enhancing therapeutic decision-making and disease monitoring, particularly in cases where higher ctDNA concentrations are present [188]. In addition, PE-NGS-cfDNA analysis of NSCLC patients is feasible and provides a comprehensive genetic test for accurate diagnosis and aids in the identification of actionable mutations in clinical practice [189].

## 5. Bioinformatic Tool Using Biomarker Analysis

**Figure 5:**
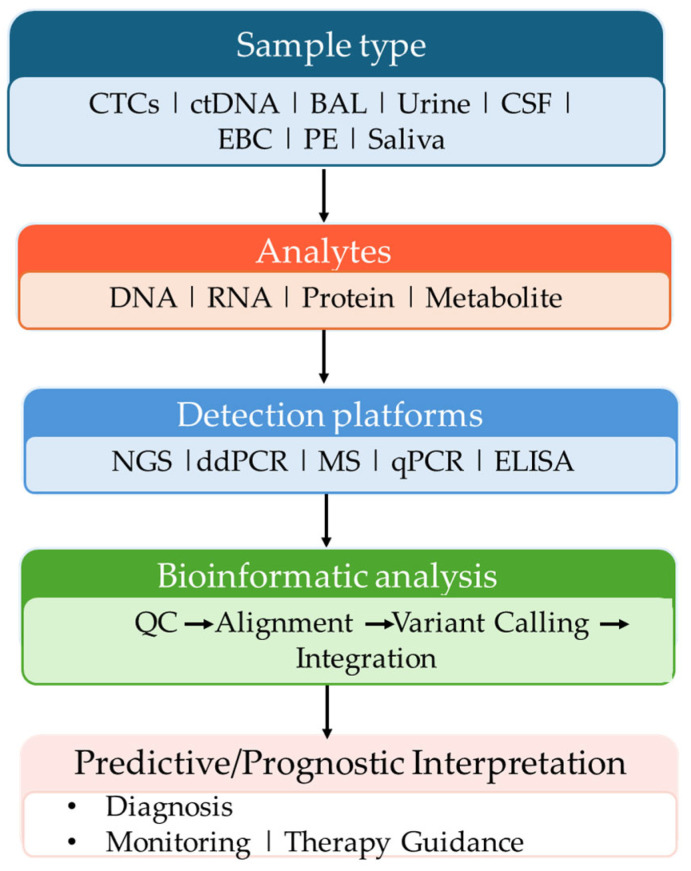
Integrated model of prognostic and predictive biomarkers in liquid biopsies.

Bioinformatics tools play a critical role in the discovery and validation of biomarkers, particularly through the analysis of large-scale, high-dimensional ‘omic’ datasets (Figure 5) [190].

For example, a copy number alteration (CNA)-based classifier built on 16 CNA profiles from CTCs in treatment-naïve blood samples of patients with SCLC successfully differentiated chemorefractory from chemosensitive cases. This classifier was also associated with a significant difference in PFS [191]. In another effort, Yuan et al. developed the HRdiffRF algorithm, which incorporates a novel hazard ratio-based splitting criterion to identify predictive biomarkers. The algorithm screened individual mutations and their combinations to support biomarker discovery for first-line chemoimmunotherapy [192].

By integrating machine learning with immunological data, dynamic changes in immune cell subsets—such as the neutrophil-to-lymphocyte ratio (NLR)—in peripheral blood have been shown to reflect alterations in the tumor microenvironment and correlate with clinical responses to immune checkpoint inhibitors [13]. A non-invasive multiparametric assay, combining ctDNA profiling with immune cell subset analysis from blood samples collected before and at the initiation of treatment, enabled early identification of patients with advanced NSCLC likely to achieve durable responses to immunotherapy [193].

Abbosh et al. introduced ECLIPSE, a bioinformatics tool designed for non-invasive tracking of subclonal tumor architecture, even at low ctDNA levels. This tool effectively identified patients with polyclonal metastatic dissemination and demonstrated that subclones destined to drive future metastases were significantly more expanded than non-metastatic subclones [190]. In a similar vein, PROPHET, developed by Chen et al., is a sensitive algorithm that identifies patients at high risk of recurrence. The model prioritizes mutations with higher allele frequency (AF), those located in driver or hotspot regions, and non-synonymous variants, offering a strategy for patient stratification and personalized treatment planning [194]. Moreover, cfDNA neomer profiling based on targeted sequencing was employed to train survival support vector machine models at various time points. This approach demonstrated superior predictive accuracy during and after treatment compared to traditional ctDNA mutation-based models [195].

## 6. Standardization of Liquid Biopsy Testing and Future Directions

The standardization of molecular testing requires consistent methodologies and reference standards to ensure accurate and comparable results across different laboratories [196]. This is particularly important for liquid biopsy testing, as only a few key molecules are often critical for managing cancer patients [197,198,199]. Inadequate analytical validation and verification can introduce bias in analytical results across laboratories, leading to overall performance that may not be reliable enough for routine patient testing [200]. The limited availability of reference materials for assessing liquid biopsy targets further poses a barrier to the validation of these tests [201].

Pre-analytical variability limits the standardization of liquid biopsy tests by introducing errors in results and significantly increasing analytical bias [202]. A key pre-analytical factor is the type of preservation tubes used, which must be carefully selected before analysis, particularly when blood samples are collected at a site distant from the clinical laboratory performing the tests [203,204]. However, the inconsistent reporting of pre-analytical variables may hinder efforts to standardize liquid biopsy testing [205]. To address these challenges, the International Society of Liquid Biopsy (ISLB) has established the Quality Control and Accreditation Committee to develop consensus-based minimal standards for ctDNA analysis in oncology [206]. Developing comprehensive quality control frameworks for pre-analytical, analytical, and interpretive processes is essential for successfully integrating liquid biopsy into routine clinical care [207].

## 7. Conclusions

In this review, we provide a comprehensive overview of the spectrum of biomarkers that can be identified through liquid biopsy across diverse biological fluids in patients with lung cancer. The inclusion of multiple sample types—such as peripheral blood, saliva, BAL, and CSF—offers an unprecedented opportunity to capture the molecular heterogeneity of the disease. Integrating these multidimensional data through advanced bioinformatics approaches enables the development of predictive algorithms and decision-support systems, ultimately guiding the implementation of precision medicine and personalized therapeutic strategies tailored to each patient’s unique molecular profile.

## Data Availability

No new data were created or analyzed in this study. Data sharing is not applicable to this article.

## Abbreviations

The following abbreviations are used in this manuscript:

SCLC	Small cell lung cancer
NGS	Next-generation sequencing
TKI	Tyrosine kinase inhibitor
OS	Overall survival
RFS	Relapse-free survival
LUSC	Squamous cell carcinoma
TMB	Tumor mutational burden
DSBs	DNA double-strand breaks
CNV	Copy number variation
WES	Whole-exome sequencing
LAF	Low allele frequency
UBL	Ubiquitin-like conjugation
ALK-I	Anaplastic lymphoma kinase inhibitor
KRT5	Keratin 5
SFTPB	Surfactant protein B
DLL3	Delta-like 3 ligand
PD	Progressive disease
BAL	Bronchoalveolar lavage
CSF	Cerebrospinal Fluid
IGHV4-4	Immunoglobulin heavy variable 4-4
AHSG	Alpha-2-HS-glycoprotein
TCGA	The Cancer Genome Atlas
NSCLC	Non-small cell lung cancer
MRD	Residual molecular disease
ctDNA	circulating tumor DNA
PFS	Progression-free survival
LUAD	Lung adenocarcinoma
HCs	Human controls
ICIs	Immune checkpoint inhibitors
VAF	Variant allele frequency
cfTL	cell-free tumor burden
MSAF	Maximum somatic allele frequency
AF	Allele frequency
SCNAs	Somatic copy-number alterations
TT	Targeted therapy
CEACAM6	CEA cell adhesion molecule 6
CTCs	Circulating tumor cells
EMT	Epithelial–mesenchymal transition
FR	Folate receptor
EBC	Exhaled breath condensate
PE	Pleural effusions
IGLV1-40	Immunoglobulin lambda variable 1-40
ECM1	Extracellular matrix protein 1
DEGs	Differentially expressed genes
TP63	Tumor protein P63
